# Factors that Enhance the Feasibility of Implementing Innovative Technology in Rural Adult Day Centers

**DOI:** 10.1093/geroni/igaf122.1709

**Published:** 2025-12-31

**Authors:** Tina Sadarangani

**Affiliations:** New York University, New York, New York, United States

## Abstract

Adult Day Services (ADS) play a critical yet underutilized role in chronic disease management and care coordination. However, communication barriers—such as reliance on paper-based systems, voicemail, and non-interoperable electronic health records—impede effective information exchange between ADS, family caregivers, and healthcare providers. This presentation introduces CareMobi™, a user-centered smartphone application designed to bridge these gaps by providing a centralized platform for family caregivers and ADS to track and share health updates, medication adherence, vital signs, and care-related documentation.A mixed methods feasibility and usability study (n = 24) was conducted in two sites (one rural, one urban) to assess CareMobi™’s impact on communication and workflow efficiency within ADS. Preliminary findings indicate that caregivers find the app intuitive and beneficial for monitoring loved ones’ well-being, facilitating real-time data sharing with ADS staff, and improving engagement with healthcare providers. Moreover, ADS staff report that CareMobi™ reduces unnecessary phone calls and enhances transparency in care coordination. This presentation will discuss study outcomes, implementation challenges and successes, and the broader implications of integrating digital tools into ADS settings. By leveraging innovative interventions like CareMobi™, ADS can enhance care continuity, caregiver confidence, and overall participant well-being.

